# Single-cell transcriptomics unveils leukocyte heterogeneity in the gills of *Larimichthys crocea* in response to parasitic infection

**DOI:** 10.3389/fimmu.2025.1633701

**Published:** 2025-08-01

**Authors:** Qiuhua Li, Meiyan Wang, Chenhao Li, Ngoc Tuan Tran, Jingqun Ao, Shengkang Li, Xinhua Chen

**Affiliations:** ^1^ Guangdong Provincial Key Laboratory of Marine Biology, Shantou University, Shantou, China; ^2^ State Key Laboratory of Mariculture Breeding, Key Laboratory of Marine Biotechnology of Fujian Province, College of Marine Sciences, Fujian Agriculture and Forestry University, Fuzhou, China

**Keywords:** gill immunity, *Larimichthys crocea*, single-cell transcriptomics, *Cryptocaryon irritans*, immune cell heterogeneity

## Abstract

**Background:**

Fish gills serve as critical immune interfaces against aquatic pathogens, yet their leukocyte heterogeneity in response to parasitic infections remains poorly understood.

**Methods:**

Single-cell RNA sequencing was employed to elucidate leukocyte responses in the gills of *Larimichthys crocea* during *Cryptocaryon irritans* infection.

**Results:**

A total of 13,070 leukocytes from the gills under steady-state and infected conditions were profiled and classified into eight principal lineages: T cells (> 70% of total immune cells), ILC2-like cells, NK-like cells, neutrophils, *cpa5* ^+^ granulocytes, B cells, macrophages, and dendritic cells. Following infection, T cell subsets exhibited distinct responses: Regulatory T cells expanded and demonstrated immunoregulatory capacity; CD8^+^ T cells exhibited cytotoxic responses; CD4^-^CD8^-^ T cells displayed Th17-like functions; and γδ T cells showed Th2-like activity. ILC2-like cells significantly increased in abundance and upregulated type 2 cytokine expression, whereas cytotoxic NK-like cells enhanced chemokine signaling and cytotoxicity. Neutrophils increased in number and oxidative activity, while *cpa5* ^+^ granulocytes highlighted immunomodulatory functions. Macrophages, dendritic cells, and B cells exhibited compartmentalized activation states, upregulating gene modules associated with pathogen recognition, antigen processing/presentation, chemotactic activity, and antibody defenses.

**Conclusions:**

These findings describe a multi-layered immune cell defense strategy in the gills of teleosts against parasitic infection, showing conserved and fish-specific adaptations. Understanding gill immunity provides viable targets for enhancing parasite resistance in aquaculture, such as modulating ILC2/Treg pathways to prevent infections.

## 1 Introduction

Vertebrates have evolved diverse respiratory structures to optimize gas exchange in their habitats (1). Aquatic species predominantly utilize evaginated structures such as gills, while terrestrial organisms employ invaginated lungs to minimize water loss. Teleost gills exhibit specialized structures: vascularized arches support filamentous projections that branch into secondary lamellae, maximizing surface area for efficient gas exchange (2). In addition to their role in respiration, gills serve as multifunctional interfaces involved in osmoregulation, acid-base homeostasis, and endocrine regulation (3–5). This direct exposure to the environment renders gills susceptible to pathogen invasion, necessitating a robust immunological system.

The gill immune system of teleosts integrates innate and adaptive components, akin to the pulmonary defenses observed in mammals (6). Subepithelial layers harbor diverse innate immune populations including monocytes/macrophages (7), granulocytes (neutrophils and mast cells/eosinophilic granule cells [MCs/EGCs]) (8), dendritic cells (DCs) (9), natural killer (NK) cells (10), and innate-like lymphocytes (ILs) (11, 12). Adaptive immunity is mediated by T cell subsets (CD4^+^, CD8^+^, and CD4^-^CD8^-^ double-negative T cells) and B lymphocytes secreting immunoglobulin isotypes (IgM, IgD, and IgT) (1, 11, 12). Although these cellular populations have been partially characterized, comprehensive molecular profiling remains challenging due to the limited availability of fish-specific antibodies, which restricts our understanding of immune cell diversity and functional specialization.

Parasitic infections pose significant threats to vertebrate health. Notably, the ciliated protozoan *Cryptocaryon irritans* causes severe cryptocaryoniasis (“white spot disease”) on gill and body surfaces, posing a major threat to marine fish aquaculture globally (13). To combat extracellular parasites (e.g., helminths), mammals depend on well-defined type 2 immune responses, characterized by the activation and expansion of various innate immune cells—such as ILC2s, basophils, eosinophils, DCs, and mast cells (14–16). A similar pattern of innate immune recruitment has been observed in teleosts during parasitic infections. For instance, in *Tinca tinca* infected with *Monobothrium wageneri*, neutrophil counts at infection sites were significantly higher than in adjacent tissues or uninfected fish (17). Teleost MCs/EGCs, which exhibit histochemical features of both mammalian mast cells and eosinophils (18), migrate and aggregate at infection sites during helminth, acanthocephalan, or cestode infections (19, 20). Recent studies highlight the role of teleost immune cells in combating *C. irritans* (13). Infection by *C. irritans* triggers the accumulation of MCs/EGCs in the secondary lamellae of the gills of *Sparus aurata* (8), as well as macrophages, neutrophils, and MCs/EGCs in *Epinephelus coioides* (21, 22). These findings indicate the critical roles of innate immune cells in fish defense against various parasites, including *C. irritans*. In addition, adaptive immune cells, including B cells and T cells, have been observed to proliferate and aggregate in gills infected by parasites, with B cells secreting parasite-specific IgM/IgT antibodies (1, 23). However, the dynamic reorganization, functional plasticity, and coordinated interplay of distinct gill immune cell subsets during an active parasitic infection remain largely unexplored, limiting our understanding of mucosal defense mechanisms.

Single-cell RNA sequencing (scRNA-seq) technologies enable high-resolution deconstruction of heterogeneous tissues and pathogen-responsive cellular states. We hypothesized that *C. irritans* infection triggers lineage-specific expansion, functional reprogramming, and the emergence of novel effector mechanisms across diverse leukocyte populations. To test this hypothesis, we applied scRNA-seq to comprehensively map the immune landscapes of *Larimichthys crocea* (large yellow croaker) gills under both steady-state and *C. irritans-*infected conditions. Our analysis revealed previously uncharacterized immune cell diversity and identified infection-induced expansions of immune cell populations, including Foxp3^+^ regulatory T cells (Tregs), neutrophils, DCs, ILC2-like cells, NK-like cells, and B cells. Furthermore, we delineated transcriptomic reprogramming associated with Treg cell differentiation, Th-like polarization, cytotoxic responses, and antibody secretion. This work establishes a fundamental atlas of gill immunity in teleosts, providing insights into anti-parasitic defense mechanisms, which have implications for disease management in aquaculture.

## 2 Materials and methods

### 2.1 Experimental fish

*L. crocea* (average weight: 50 g) were obtained from a mariculture farm in Ningde, Fujian, China. Fish were acclimated in 100-L fiberglass-reinforced plastic tanks equipped with a recirculating aquaculture system (24 ± 2°C, UV-sterilized seawater) and fed daily with commercial pellets. After a 14-day acclimatization period, healthy individuals were selected for subsequent experiments. All procedures adhered to the Laboratory Animal Guideline (GB/T 35892–2018) and were approved by the Animal Ethics Committee of Fujian Agriculture and Forestry University. All fish were anesthetized using tricaine methanesulfonate (MS-222; MCE) before surgical dissection.

### 2.2 *C. irritans* challenge

*C. irritans* tomonts were harvested from naturally infected *L. crocea* and maintained in sterile seawater at 8°C with daily water renewal (23). The excystation of tomonts was induced by transferring them to 26°C for 72 hours. Freshly released theronts, less than 2 hours post-hatching, were quantified and used to infect fish. The experimental group was challenged with 3,000 theronts per fish through a 2-hour static immersion, while the control group underwent identical handling procedures without parasite exposure. Following the challenge, fish were transferred to separate flow-through tanks maintained at 24 ± 2°C. Gill tissues were collected at 60 hours post-infection (peak trophont maturation phase) for leukocyte isolation.

### 2.3 Leukocyte isolation

Gills were aseptically dissected, minced into 1-mm^3^ fragments, and digested in cold DMEM containing 1 mg/mL collagenase I, 1 mg/mL collagenase IV, 0.1% (w/v) NaCl, and 1× penicillin/streptomycin at 4°C for 2 hours. Tissue homogenates were filtered through a 70-μm cell strainer (BD Biosciences), washed twice with DMEM supplemented with 2% fetal bovine serum (FBS), and separated using a 34% Percoll gradient by centrifugation at 650 × *g* for 30 minutes at 4°C. Pelleted cells were treated with red blood cell (RBC) lysis buffer, washed, and assessed for viability (>90% by trypan blue exclusion). Cell counts were quantified via flow cytometry.

### 2.4 Single-cell RNA sequencing

Cell suspensions from the gills of five fish per group were pooled and adjusted to a concentration of ~1,000 cells/μL. These preparations were processed using the Chromium™ Single Cell 3’ Kit (10× Genomics). Gel bead-in-emulsions generation, cDNA amplification, and library preparation were performed following the manufacturer’s protocols. Libraries were sequenced on an Illumina NovaSeq 6000 platform (150-bp paired-end) by Gene Denovo Biotech (Guangzhou, China).

### 2.5 scRNA-seq data processing and cell type identification

Raw sequencing data were aligned to the *L. crocea* reference genome (GCF_000972845.2) using Cell Ranger (v3.1.0). Doublets were removed using DoubletFinder (v2.0.3). The cells were excluded if they met any of the following criteria: <500 or >4,000 detected genes, >20,000 UMI counts, or >10% mitochondrial reads. Post-filtering, data were normalized, integrated, and clustered using Seurat (v4.0.4) with 30 principal components at a clustering resolution of 0.5. Cell clusters were visualized using Uniform Manifold Approximation and Projection (UMAP). Cell identities were annotated based on conserved immune cell markers.

### 2.6 Differential expression analysis

For comprehensive identification of cluster-specific markers across all cellular subsets, the Wilcoxon rank-sum test was implemented through the FindAllMarkers function, comparing each cluster against the rest cells from all other clusters. Significantly upregulated genes were identified based on the following criteria: (1) genes had to be expressed at least 1.28-fold higher in the target cluster compared to other clusters; (2) genes must be detected in more than 25% of the cells within the target cluster; (3) an unadjusted *p*-value < 0.05. To investigate condition-specific transcriptional changes within defined immune subclusters, intra-cluster differential expression analysis was performed between control and infected groups using a hurdle model in MAST (Model-based Analysis of Single-cell Transcriptomics). Differentially expressed genes (DEGs) were defined as meeting all of the following thresholds: (1) absolute log_2_-fold change (log_2_FC) ≥ 1; (2) adjusted *p*-value ≤ 0.05 (Benjamini-Hochberg correction); (3) detection in ≥ 20% of cells within the target cluster.

### 2.7 Pathway enrichment

DEGs were subjected to Gene Ontology (GO) and Kyoto Encyclopedia of Genes and Genomes (KEGG) enrichment analysis using a hypergeometric test with false discovery rate (FDR) correction (FDR < 0.05). Enriched terms were mapped to biological processes, molecular functions, or pathways.

## 3 Results

### 3.1 Comprehensive immune cell annotation revealed T cell dominance in teleost gill immunity

**Figure 1:**
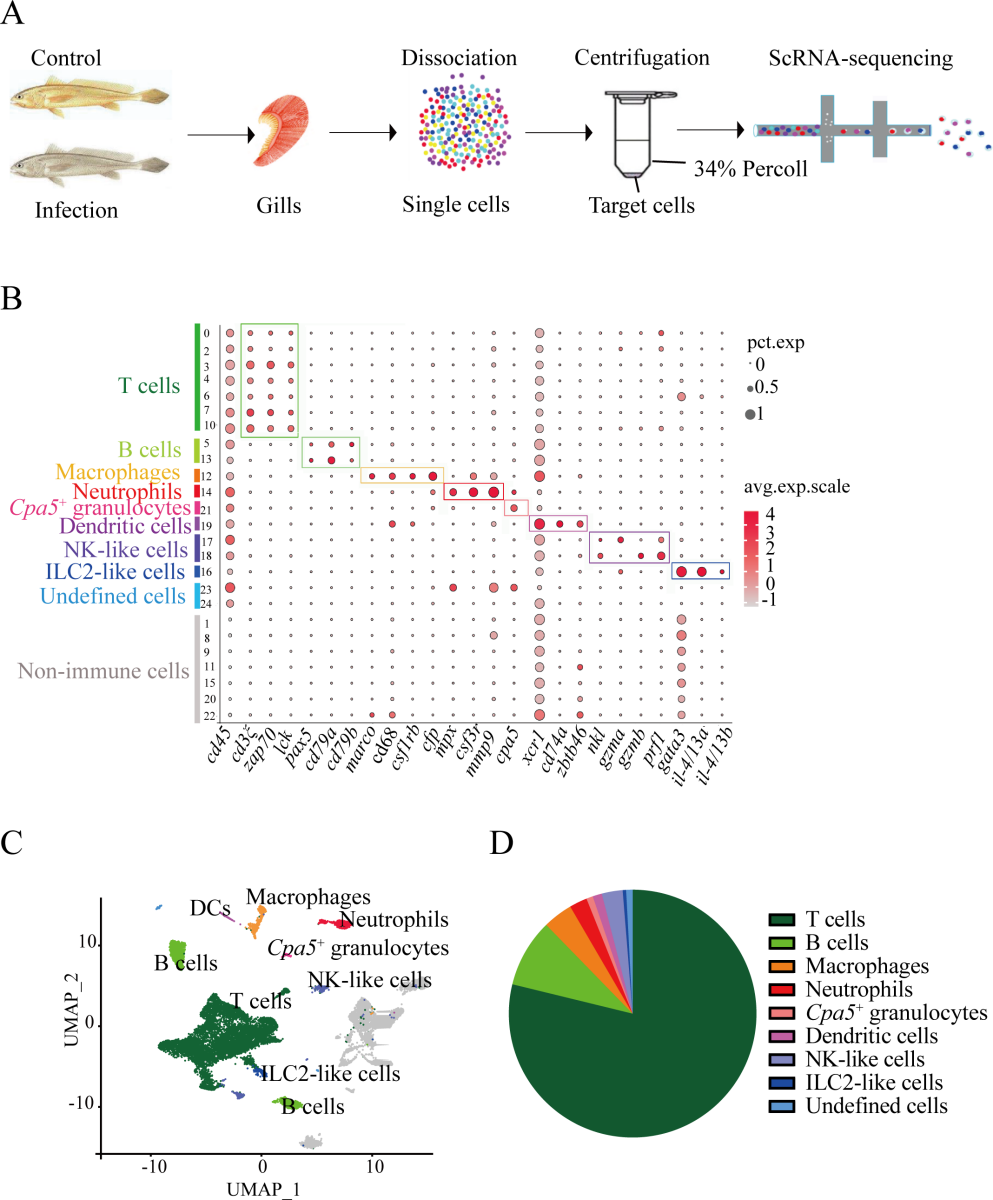
Major immune cell types in the *L. crocea* gills identified by 16,832 single-cell transcriptomes. **(A)** Schematic of experimental design. **(B)** Dot plot of putative marker genes for T cells, B cells, macrophages, neutrophils, *cpa5* ^+^ granulocytes, dendritic cells (DCs), ILC2-like cells, NK-like cells, and non-immune cells across clusters. Bubble size and color intensity indicate the percentage of cells expressing each gene and the average expression level per cluster, respectively. **(C)** UMAP visualization of all immune cells. **(D)** Pie chart showing the relative abundance of major immune cell types in gills.

To delineate the immune landscape of *L. crocea* gills, we conducted comparative scRNA-seq profiling of leukocytes isolated from control and *C. irritans*-infected specimens (**Figure 1A**). Sequencing achieved an average of 31,660 reads/cell (997 genes/cell) and 37,067 reads/cell (980 genes/cell) in the control and infected samples, respectively. Following stringent quality control (**Supplementary Figure S1**), 16,832 high-quality transcriptomes (control: 9,028; infected group: 7,804) were retained for further analysis. Unsupervised clustering delineated 25 transcriptionally distinct cell states, validated through UMAP visualization and cluster-specific marker heatmaps (**Supplementary Figure S2**).

The 25 clusters were annotated into eight immune lineages and non-immune contaminants (**Figures 1B, C**; markers in **Supplementary Table S1**). T lymphocytes occupied clusters 0, 2, 3, 4, 6, 7, and 10, defined by the expression of canonical T-cell markers *cd3ξ*, *zap70*, and *lck*. B-cell compartments comprised clusters 5 (B-5) and 13 (B-13), identified by *pax5*, *cd79a*, and *cd79b*. Myeloid populations, including macrophages (Cluster 12 with the expression of *csf1rb* ^+^/*cfp* ^+^/*macro* ^+^/*cd68* ^+^), neutrophils (Cluster 14: *mpx* ^+^/*csf3r* ^+^/*mmp9* ^+^), DCs (Cluster 19: *cd74a* ^+^/*xcr1* ^+^/*zbtb46* ^+^), and *cpa5* ^+^ cells (Cluster 21: *cpa5^+^*), were identified. Notably, *cpa5* is specifically expressed in the zebrafish mast cell lineage (24). However, multiple mast cell markers (including *kita* and *tps*) were not detected (data not shown) in *cpa5* ^+^ cells. These suggest that these cells can be classified as *cpa5* ^+^ granulocytes and potentially represent a teleost-specific granulocyte subset with partial functional overlap with mast cells. Innate lymphoid diversity spanned NK-like cells (Clusters 17-18: *nkl* ^+^/*gzma* ^+^/*gzmb* ^+^), and ILC2-like cells (Cluster 16: *gata3* ^+^/*il4/13a* ^+^/*il4/13b* ^+^). A total of 3,762 non-immune cells (Clusters 1, 8, 9, 11, 15, 20, and 22), which lacked *cd45* expression, were excluded from immune analyses. Transcriptomic analysis identified the top 20 differentially expressed genes per immune lineage, revealing both evolutionarily conserved and teleost-specific immune signatures (**Supplementary Table S2**). Strikingly, T cells constituted 71.6% (9,360 cells) of immune cell population (**Figure 1D**; **Supplementary Table S3**), establishing their central role in piscine mucosal immunity. In mammals, T cells comprised functionally distinct subsets, which mediate specialized roles in response to the parasite infection (25). This dominance provides the cellular substrate for exploring the diverse T cell subset responses (proliferation, differentiation, and cytokine production) during *C. irritans* infection.

### 3.2 Heterogeneity of T cell subsets

**Figure 2:**
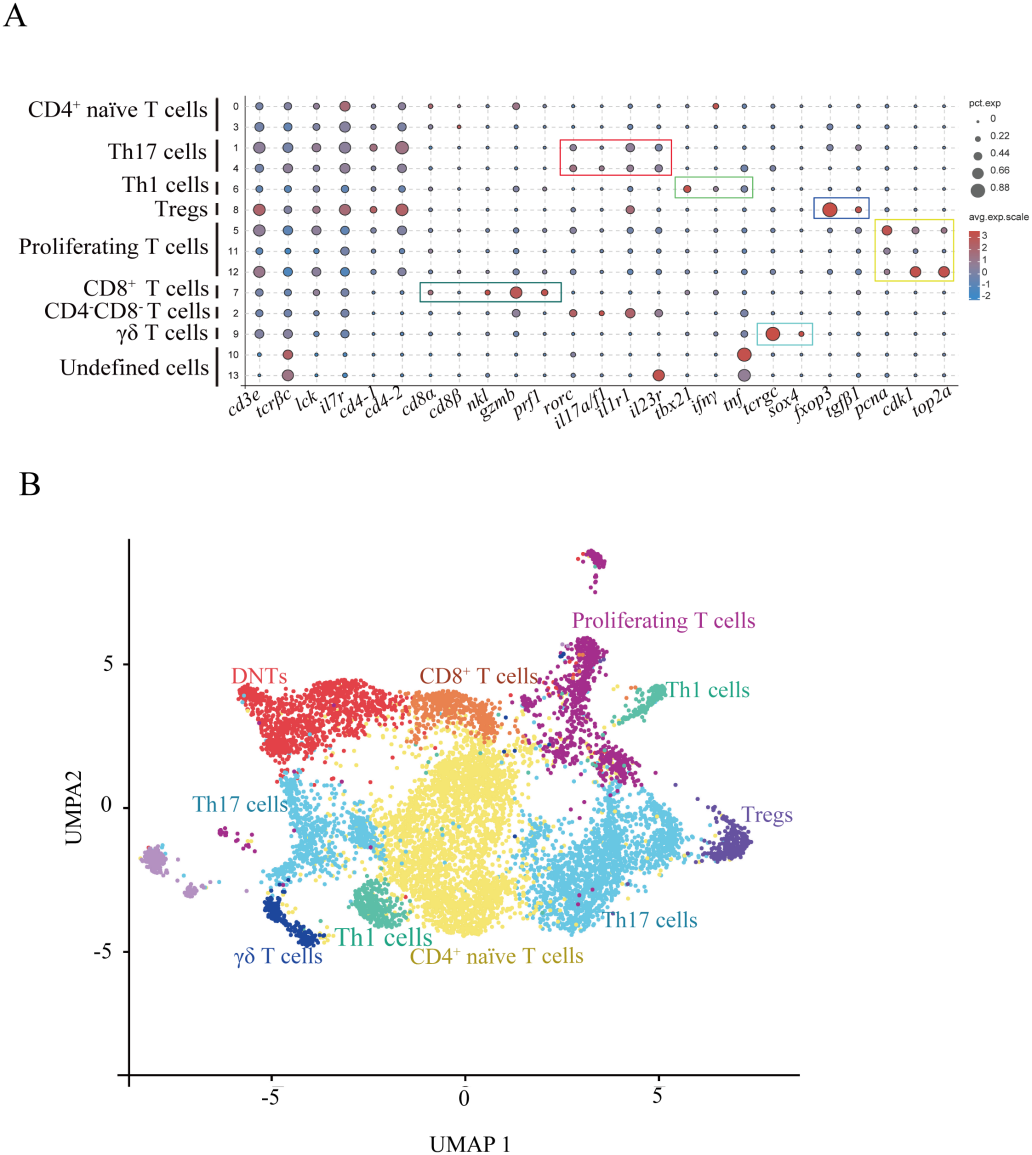
Heterogeneous subsets of T cells in gills. **(A)** Dot plots displaying marker gene expression levels and proportions for each T cell subset. **(B)** UMAP visualization of T cell subsets.

Our analysis partitioned T cells from two biological samples into 14 clusters (designated T0–T13) using a clustering resolution of 0.5 (**Supplementary Figure S3A**). Cluster validation through visualization of the top five variably expressed genes revealed a clear demarcation between all subsets in the heatmap analysis (**Supplementary Figure S3B**). Differential gene expression profiles across clusters, integrated with typical T-cell lineage markers, allowed for annotation of definitive subsets (**Figure 2**; DEGs in **Supplementary Table S4**; markers in **Supplementary Table S5**). The cluster T7 was classified as CD8^+^ cytotoxic T lymphocytes (CTLs) by expressing *cd8a*, *cd8β*, *nkl*, *gzmb*, and *prf1* (**Figure 2A**). CD4^+^ T cell populations segregated into four functionally distinct subgroups, including regulatory T cells (Tregs), Th17 cells, Th1 cells, and naïve T cells (**Figures 2A, B**). The results found that the cluster T8 was annotated as Tregs (expressing *foxp3* and *tgfb1*), T6 as Th1 cells (expressing lineage-defining transcription factor *tbx21* and effector cytokines *ifng* and *tnfa*), T1 and T4 as Th17 cells (expressing characteristic markers *rorc* (RORγt), *il17a/f1*, *il1r1*, and *il23r*), T9 as γδ T cells (expressing *tcrgc* and a transcription factor *sox4* (26), lacking CD4 and CD8 receptors), T2 as CD4^-^CD8^-^ double-negative T cells (DNTs; lacking CD4 and CD8 expression), T5, T11, and T12 as proliferating T-cell subset (expressing DNA replication genes *pcna*, *cdk1*, and *top2a*). The ten most statistically significant subset-defining genes across all T cell populations showed the potential markers for T cell subsets in fish (**Supplementary Table S6**).

### 3.3 *C. irritans* challenge induced T cell proliferation and CD4^+^ T cell differentiation in gills

**Figure 3:**
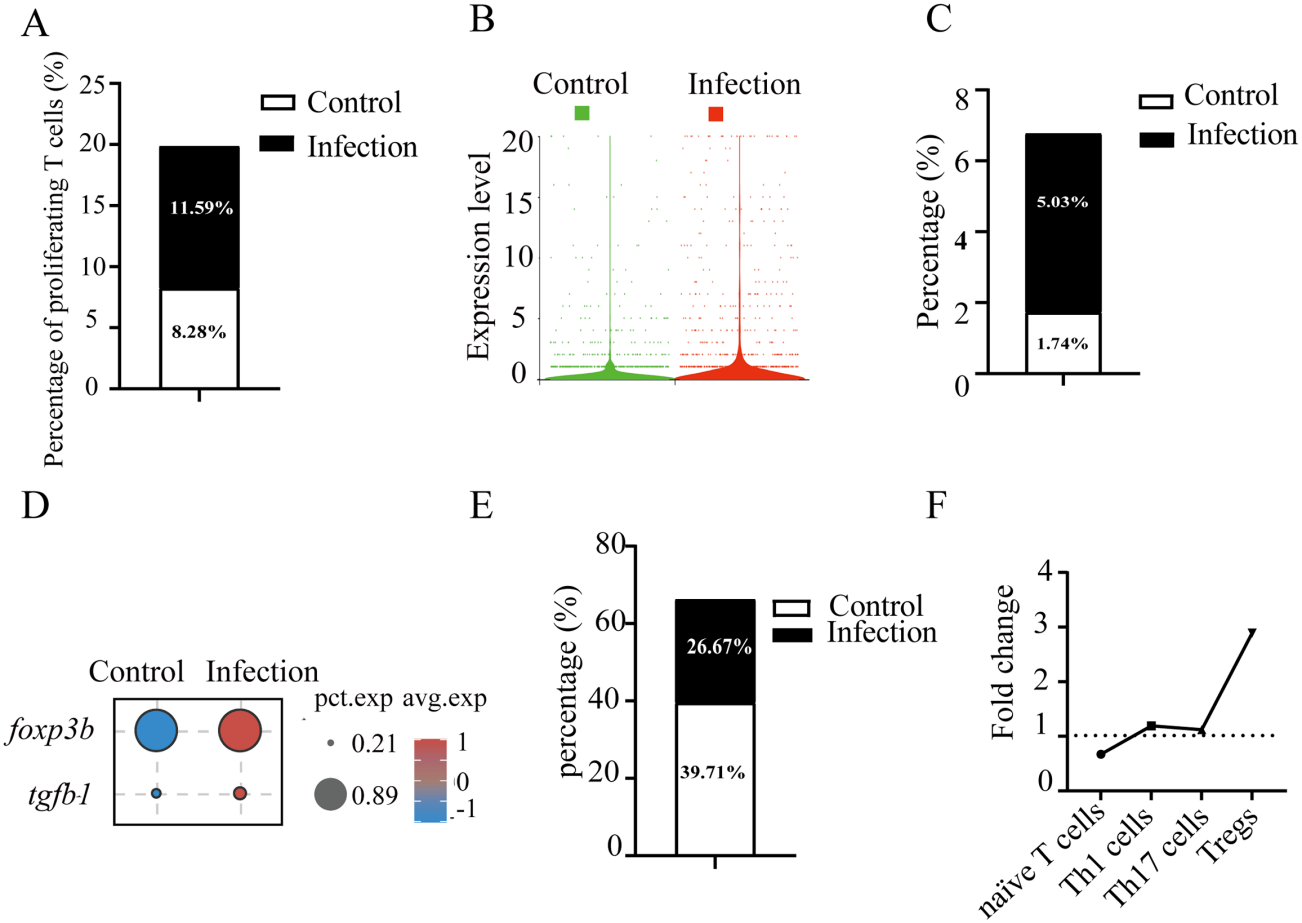
C. *irritans* infection induced T cell proliferation and Foxp3^+^ Treg differentiation. **(A)** Proportions of proliferating T cells (as a percentage of total T cells) in *C*. *irritans*-infected vs. normal gill tissues. Similar subpopulation analyses were performed for Tregs **(C)** and CD4^+^ naïve T cells **(E)**. **(B)** Elevated average expression of *pcna* in the *C*. *irritans*-infected group compared to the control group. **(D)** Dot plots displaying expression levels of Treg cell-associated markers and the proportion of cells. **(F)** Line chart depicting fold changes in the percentages of CD4^+^ naïve T cells, Th1 cells, Th17 cells, and Tregs post-*C. irritans* infection.

To investigate the T cell-mediated immune responses in gill tissue during pathogen challenge, we first assessed the redistribution of T cell subsets after *C. irritans* infection. At 60 hours post-infection, the number of proliferating T cells (T5, 11, and 12) increased from 8.28% to 11.59% (**Figure 3A**). Concurrently, an upregulation of *pcna* (encoding the proliferating cell nuclear antigen that enhances DNA polymerase processivity during replication) was observed in infected fish (**Figure 3B**). These data demonstrate the induction of T cell proliferation in gill tissue by *C. irritans* infection. Although overall T cell number decreased, mainly due to a sharp increase in myeloid cells, Treg numbers increased 2.89-fold (from 1.74% to 5.03%) after infection (**Figure 3C**). This Treg amplification was supported by significant induction of lineage-defining markers *foxp3* and *tgfb1* following pathogen exposure (**Figure 3D**). Furthermore, the proportion of naïve T cells (T0 and T3) decreased significantly, from 39.71% to 26.67% of total T cells (**Figure 3E**). Notably, Th1 and Th17 subsets remained stable after infection (**Figure 3F**), supported by the unchanged expression of their signature transcriptional regulators (*tbx21* and *rorc*) and effector cytokines (*ifng* and *il17a/f1*). These results suggest preferential differentiation of naïve T cells into immunosuppressive Tregs rather than effector subsets during *C. irritans* infection.

### 3.4 DNTs in gills exhibited Th17-like function

**Figure 4:**
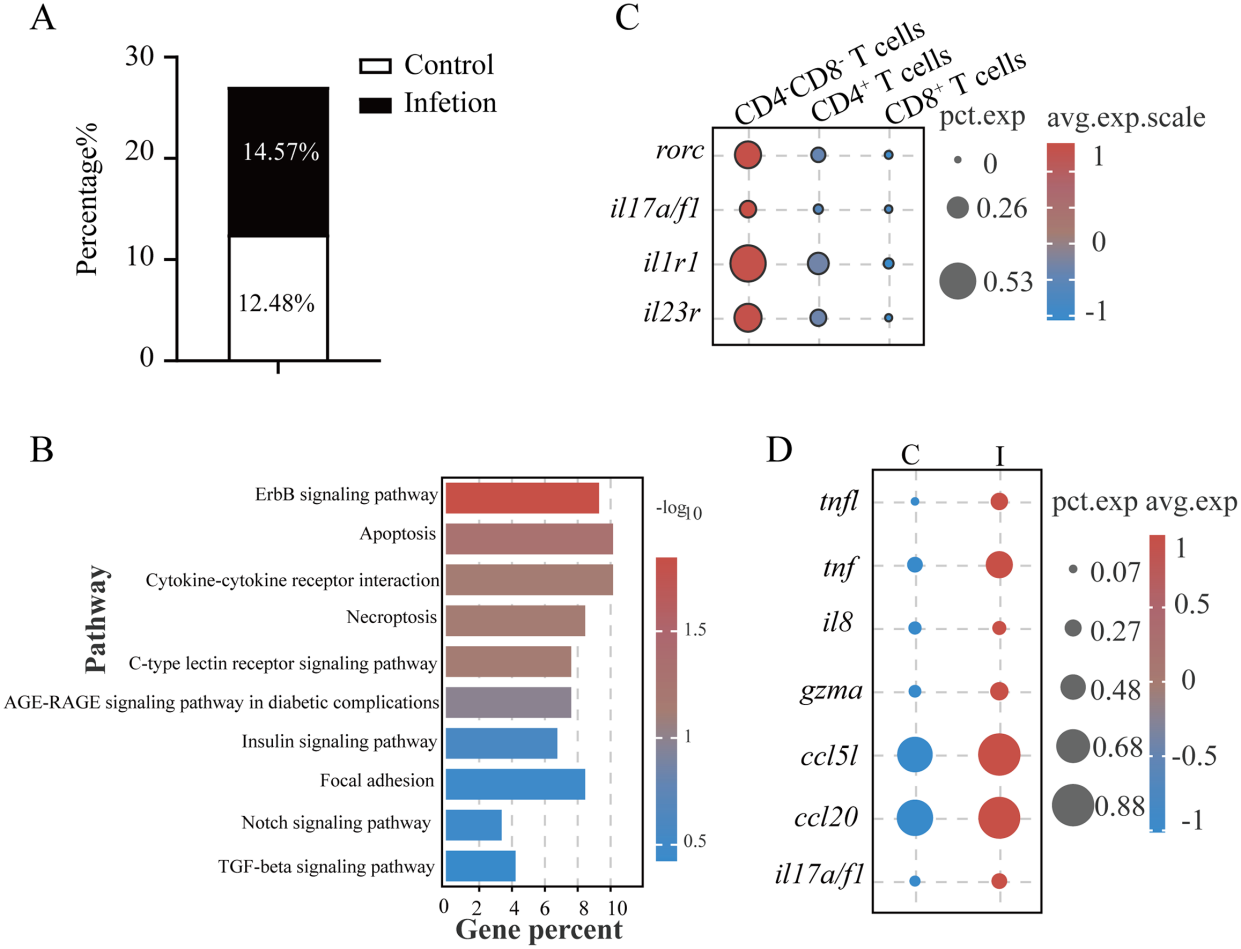
DNTs in *L. crocea* possessed Th17-like functions. **(A)** Proportions of DNTs in total T cells. **(B)** KEGG pathway enrichment in DNTs. **(C)** Th17-associated marker expression profiles in DN (CD4^–^CD8^–^), CD4^+^ and CD8^+^ T cells. **(D)** Immune-related gene expression in DNTs from control (C) and infected (I) groups.

DNTs exhibit functional plasticity through cytokine secretion (IL-4, IL-17, IFNγ, and TNFα) mirroring the activities of CD4^+^ T helper cells, termed helper DN T cells or Th-like DN T cells (27, 28). These functional DNTs demonstrate context-dependent protective or pathogenic roles across infection models (27, 29). Our results revealed that *L. crocea* DNTs (*Lc*DNTs) accounted for 12.48% of total gill T cells under homeostasis, increasing to 14.57% after *C. irritans* challenge (**Figure 4A**). Comparative transcriptomic profiling across CD4^+^, CD8^+^, and DNT populations through integrative analysis identified subset-defining molecular signatures (**Supplementary Figure S4A**). Gene ontology analysis revealed DNT-specific enrichment of regulatory pathways, particularly metabolic process modulation and systemic biological regulation (**Supplementary Figure S4B**). *Lc*DNTs were prominently engaged in cytokine-cytokine receptor and C-type lectin signaling pathways, as evidenced by differential expression of key mediators including cytokine receptors (*il2rb*, *il1r1*, and *il17rα*), death receptors (*dr4*), chemotaxis regulators (*ccr6* and *ccl20a.3*), inflammatory cytokines (*il22* and *tnf*), and signaling components (*fcrγ*, *mapk1*, *pik3r1*, and *ikba*) (**Figure 4B**, **Supplementary Table S7**). Notably, *Lc*DNTs displayed Th17-polarized features, characterized by the elevated expression of lineage-defining transcription factor (*rorc*) and (molecules *il17a/f1*, *il1r1*, and *il23r*) compared to conventional CD4^+^ and CD8^+^ T cells (**Figures 4C**, **2A**). Parasitic challenge induced the expression of *il17a/f1* production (**Figure 4D**), as well as cytotoxic effector (*gzma*) and immunomodulatory factors (*ccl5l*, *ccl20*, *il8*, *tnf*, and *tnfl*) in *Lc*DNTs (**Figure 4D**; **Supplementary Table S8**), confirming a functional Th17-like programming and dual cytotoxic and regulatory capacities, respectively. Systematic analysis of alternative Th-like DNT subtypes (e.g., Th1 and Th2) demonstrated minimal expression of lineage-specifying transcription factors (*tbx21* and *gata3*), supporting a predominant Th17-like polarization phenotype.

### 3.5 Characterization of γδ T cells and CD8^+^ T cells

**Figure 5:**
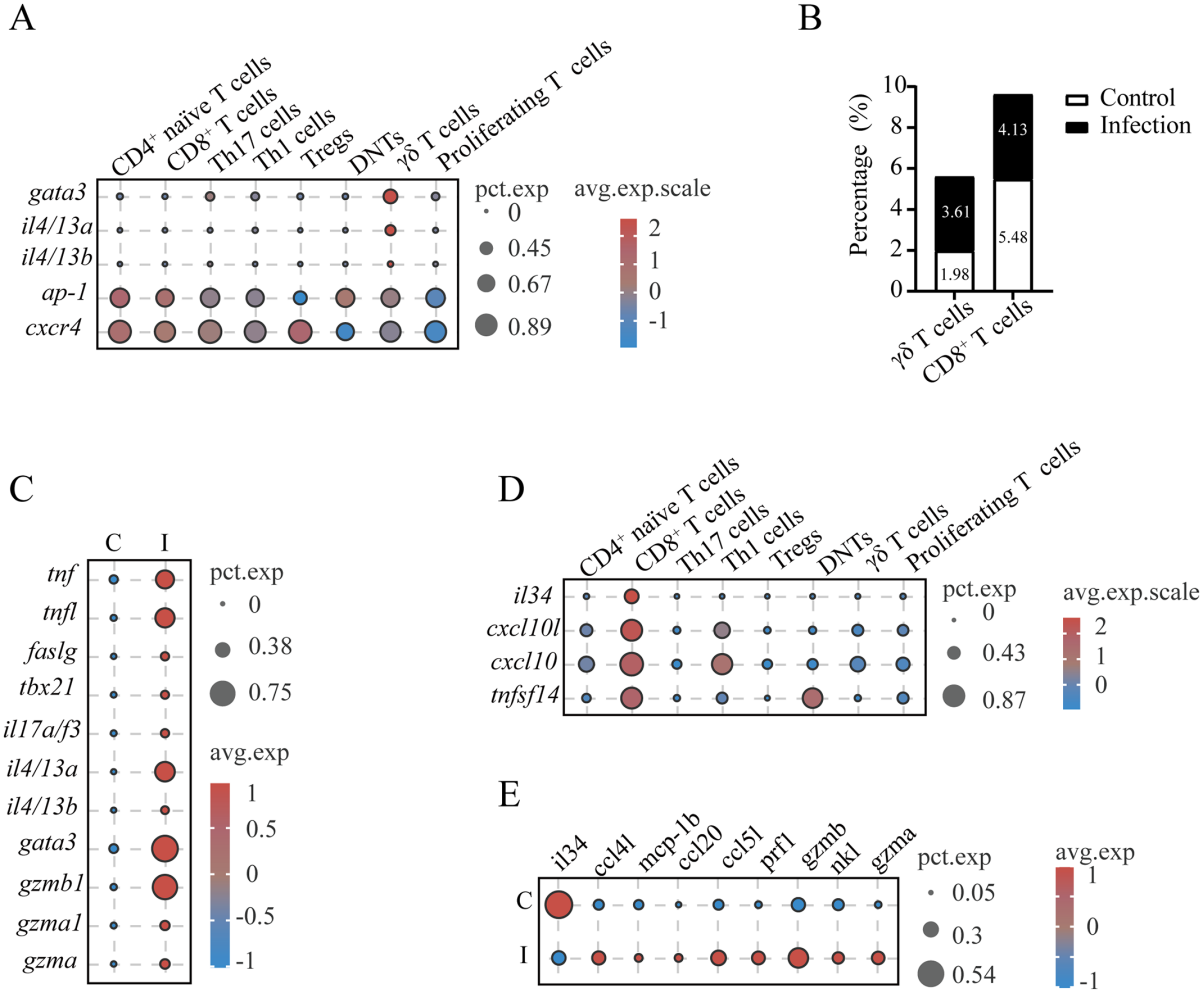
Functional characterization of γδ T cells and CD8^+^ T cells in *L. crocea* gills. **(A)** Expression profiles of type 2 immune polarization-associated genes in γδ T cells of *L. crocea*. **(B)** Proportional abundance of γδ T cells and CD8^+^ T cells relative to total T cells across experimental groups. **(C)** Comparative expression profiles of immune-related genes in γδ T cells between control (C) and *C*. *irritans*-infected (I) groups. **(D)** Cytokine expression signatures in CD8^+^ T cell populations. **(E)** Differential expression patterns of immune-related genes in CD8^+^ T cells from control versus infected groups.

In mammalian systems, γδ T cells represent a functionally heterogeneous lymphocyte population with specialized roles in antimicrobial defense and immunomodulation. These cells exhibit remarkable plasticity, differentiating into cytotoxic effectors and helper-like subsets (Th1/Th2/Th17 analogs) upon activation while producing diverse cytotoxic mediators and cytokines to modulate the immune response (30). Our results identified *L. crocea* γδ T cells (cluster T9) as multifunctional effectors, demonstrating enriched expression of chemotaxis regulators (e.g., *cxcl10*, *mcp1b*, and *ccl3*), endocytosis and antigen processing components (e.g., *cxcr4*, *chmp4b*, *arf6*, *cd74*, *h2-q9*, *rt1-b*, and *hla-drα*), cytotoxic effectors (e.g., *gzmb*), and inflammatory mediators (e.g., *tnf*, *tnfl*, and *il17a/f3*) (**Supplementary Tables S9**, **S10**). Unique adhesion molecules *cd2l* and *cldn4* (claudin-4) were specifically expressed in γδ T cells, potentially mediating intercellular interactions within gill microenvironments (**Supplementary Tables S9**, **S10**). The data revealed type 2 immune polarization in *L. crocea* γδ T cells, as evidenced by elevated expression of *gata3*, *ap-1*, *cxcr4*, and type 2 cytokines (*il4/13a* and *il4/13b*, homologous to mammalian *il13* and *il4*, respectively) (**Figure 5A**). Parasitic challenge triggered a significant increase in the number of γδ T cells, ranging from 1.98% to 3.61% of total T cells (**Figure 5B**). Results also found that γδ T cells in the gills of controls exhibited minimal production of cytokines (such as *tnf*, *il17a/f3*, and *tnfl*). In contrast, the upregulation of cytotoxic effectors (*gzma* and *gzmb*), Th1/Th17-polarizing cytokines (*tnf*, *tnfl*, *faslg*, and *il17a/f3*), and Th2-associated transcriptional regulators (*gata3*) and cytokines (*il4/13a* and *il4/13b*) was found in infected fish (**Figure 5C**).

Furthermore, the CD8^+^ CTL population (cluster T7) was found to express cytolytic effectors (*nkl*, *gzmb*, *gzma*, and *prf1*), immunoregulatory cytokines (*il34* and *tnfsf14*), and chemoattractant *cxcl10* (**Figures 2A**, **5D**). Although the proportion of CD8^+^ CTLs decreased from 5.48% to 4.13% after infection (**Figure 5B**), these cells exhibited enhanced expression of cytolytic genes (*nkl*, *gzmb*, *gzma*, and *prf1*) and chemokines (*ccl5l*, *ccl20*, *mcp1b*, and *ccl4l*), while decreased the expression of *il34* (**Figure 5E**). This dichotomy suggests CD8^+^ CTLs balance direct cytotoxicity (*gzmb*, *gzma*, and *prf1*) with immunoregulatory chemokine signaling during anti-parasitic responses.

### 3.6 *Lc*ILC2-like cells in gills exerted the Th2-like function upon *C. irritans* infection

**Figure 6:**
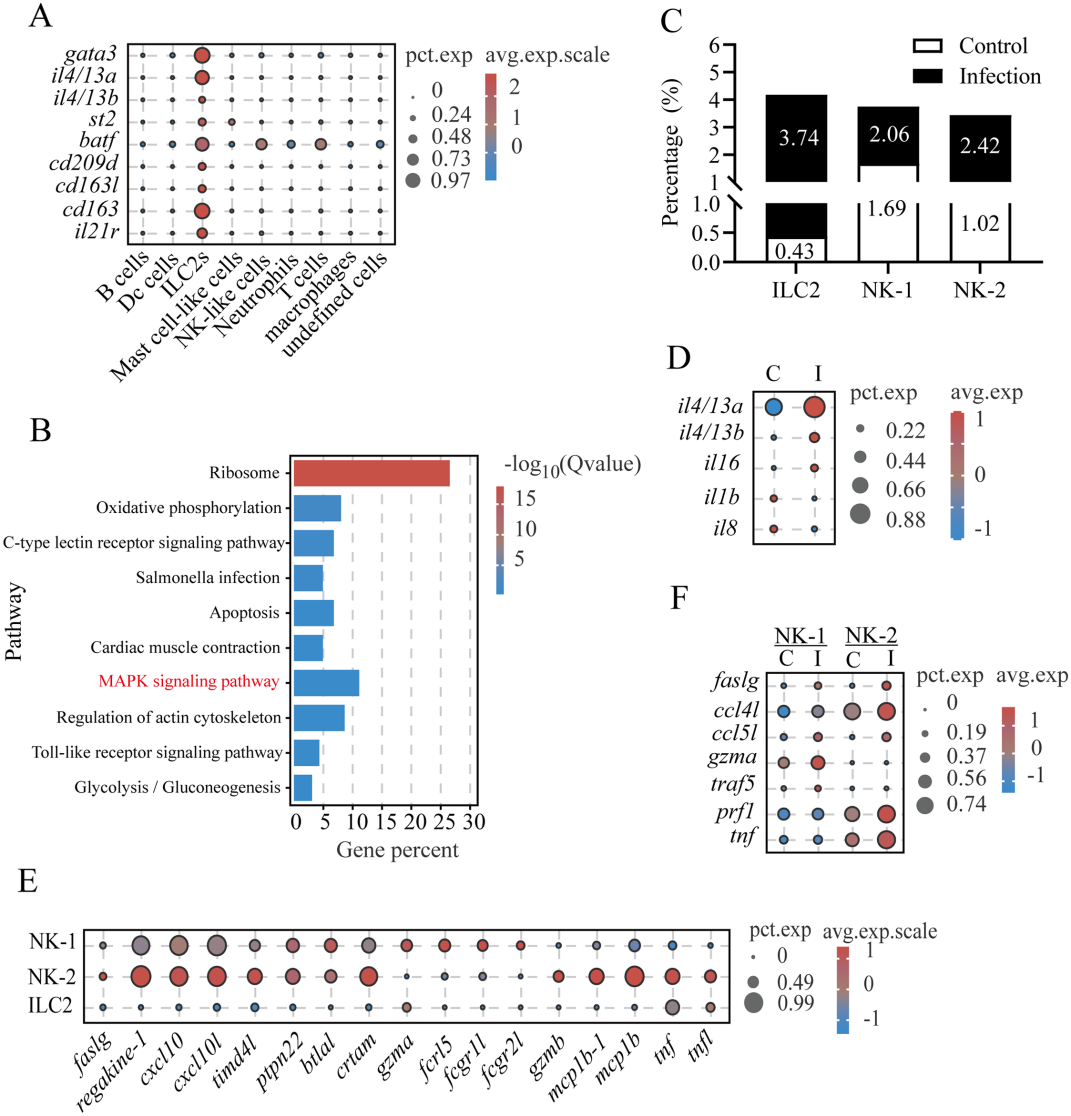
*L. crocea* ILC2-like cells exerted Th2-like functions upon *C*. *irritans* infection. **(A)** ILC2-like cell marker expression profiles. **(B)** KEGG pathway enrichment in ILC2-like cells. **(C)** Proportions of ILC2-like, NK-1, and NK-2 cells in total immune cells. **(D)** Immune-related gene expression in ILC2-like cells (control vs. infected). **(E)** Shared and differentially expressed genes between NK-1 and NK-2 subsets. **(F)** Infection-induced changes in NK-1 and NK-2 immune-related genes.

A set of genes highly or specifically expressed in *Lc*ILC2-like cells (*Lc*ILC2s) was identified across samples, establishing a core transcriptomic signature for ILC2 populations in teleost gills (**Supplementary Table S11**), including transcription factor *gata3* (essential for ILC2 lineage development) (31) and key effector cytokines *il4/13a* and *il4/13b* (**Figures 1B**, **6A**; **Supplementary Table S12**). Notably, these cells expressed the *st2* (*il1rl1*, encoding the IL-33 receptor), a hallmark surface marker for ST2^+^ ILC2s, and *batf*, a crucial regulator of IL-25-responsive migratory ILC2 cell fate and function (**Figure 6A**) (32, 33). Conservation of intracellular signaling was demonstrated through enrichment of MAPK pathway components (*flt3*, *dusp5*, *mapk1*, *myd88*, *mknk1*, and *mras*), which control proliferation and cytokine production in mammalian ILC2s (**Figure 6B**) (34–36). Intriguingly, typical mammalian ILC2-activating receptors (*il-7r*, *il-9r*, *tslpr*, and *il25r*) (36) were undetectable in *Lc*ILC2s, potentially reflecting teleost-specific adaptations. Instead, this population uniquely expressed endocytic receptors (*cd209d*, *cd163l*, and *cd163*), along with cytokine receptor (*il21r*) (**Figure 6A**). Following parasitic challenge, *Lc*ILC2s underwent an 8.7-fold expansion (from 0.43% to 3.74% of gill leukocytes; **Figure 6C**). The infection activated the upregulation of cytokine expression (*il4/13a*: 2.6-fold; *il4/13b*: 5.4-fold), and downregulation of *il1b* and *il8* (**Figure 6D**).

Within innate lymphoid populations, two cytotoxic NK cell subsets (NK-1 in cluster 17 and NK-2 in cluster 18) were characterized. Both subsets expressed core effector molecules (*nkl*, *faslg*, and *prf1*), chemotactic factors (e.g., *regakine-1* and *cxcl10*), and immunoregulatory properties (e.g., *timd4l*, *ptpn22*, *btlal*, and *crtam*) (**Figures 1B**, **6E**). NK-1 mainly expressed *gzma* and Fc receptor-related genes (*fcrl5*, *fcgr1l*, and *fcgr2l*), whereas NK-2 expressed *gzmb* and cytokines (*mcp1b, tnf*, and *tnfl*) (**Figure 6E**). This transcriptional divergence suggests compartmentalized functional roles between the cell subsets. The infection increased the proportion of both NK-1 (from 1.69% to 2.06%) and NK-2 (from 1.02% to 2.42%) (**Figure 6C**) and upregulated the pro-apoptotic effector (*faslg*) and chemokines (*ccl5l* and *ccl4l*) (**Figure 6F**). Subset-specific responses included an elevated expression of *gzma* and *traf5* in NK-1 compared with *prf1* and *tnf* upregulation in NK-2 (**Figure 6F**). These differential profiles suggest distinct cytotoxic mechanisms and compartmentalized immunoregulatory roles for NK-1 and NK-2 during *C. irritans* infection.

### 3.7 Differences of two granulocytes in gills and their response to *C. irritans* infection

**Figure 7:**
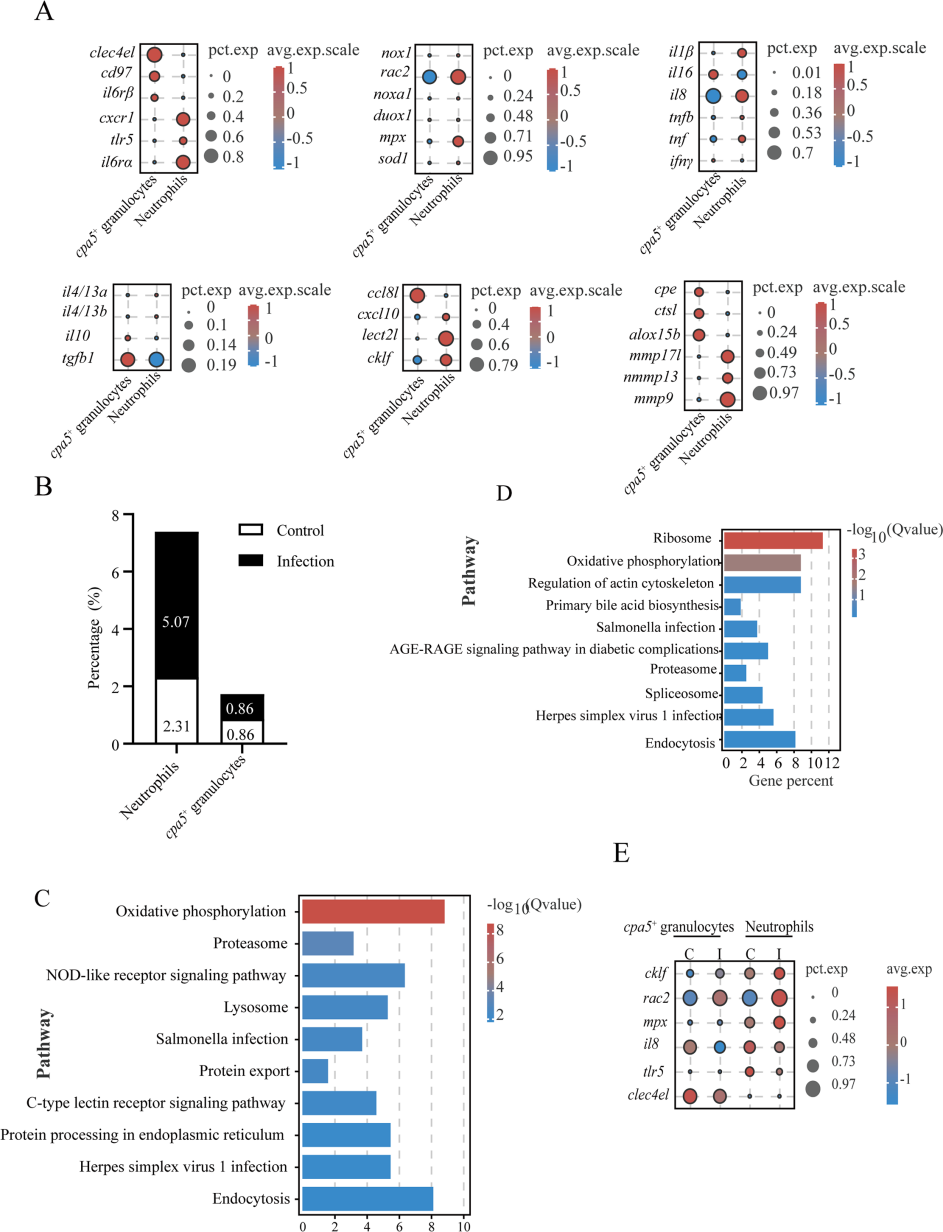
Comparative analysis of granulocyte populations and their responses to *C*. *irritans* infection. **(A)** Dot plot of differentially expressed genes between neutrophils and *cpa5* ^+^ granulocytes. **(B)** Neutrophil and *cpa5* ^+^ granulocyte cellular proportions in control vs. infected groups. **(C, D)** KEGG pathway enrichment in neutrophils **(C)** and *cpa5* ^+^ granulocytes **(D)**. **(E)** Immune-related gene expression changes in neutrophils and *cpa5* ^+^ granulocytes post-infection.

Granulocytes are considered to be important effector cells in the protection of the gill mucosa of teleosts (8). However, the specific roles of individual subsets during parasitic infection are not well understood. To address this gap, we compared neutrophils and *cpa5* ^+^ granulocytes to elucidate their functions in the gills of *L. crocea* during *C. irritans* challenge. Among 17,781 detected genes, 549 DEGs were identified in neutrophils, compared to 243 DEGs in *cpa5* ^+^ granulocytes. GO enrichment analysis revealed that neutrophils were predominantly associated with proton transport (GO:0006818), hydrogen transport (GO:0015992), and macromolecular complex assembly (GO:0034622) (**Supplementary Figure S5A**). The *cpa5* ^+^ granulocytes exhibited enrichment in processes related to single-organism signaling (GO:0044700), signaling receptor activity (GO:0023052), and cell communication (GO:0007154) (**Supplementary Figure S5B**). KEGG pathway analysis revealed significant enrichment of phosphatidylinositol signaling (ko04070), insulin signaling (ko04910), and C-type lectin receptor signaling pathways (immune recognition) in *cpa5* ^+^ granulocytes, while oxidative phosphorylation (ko00190), proteasome activity (ko03050), phagocytosis (ko04145), and endocytosis (ko04144) pathways in neutrophils (**Supplementary Figures S5C, D**). In addition, *cpa5* ^+^ granulocytes are involved in the expression of immune-related genes (such as *clec4el*, *il6rβ*, and *cd97*) and immunoregulatory factors (*ccl8l*, *il16*, and *tgfb1*), while neutrophils are related to the expression of chemotaxis receptors (*cxcr1*), inflammatory mediators (*il1β* and *il8*), chemokines (*cklf* and *lect2l*), and respiratory burst components (*rac2*) (**Figure 7A**; **Supplementary Table S13**). Additionally, protease profiling revealed lineage-specific enzymatic arsenals (*mmp17l*, *mmp13*, *mmp9*, *cpe*, *ctsl*, and *alox15b*), further supporting their different roles in immune defense (**Figure 7A**). These findings highlight significant functional differences between the two granulocyte populations, with neutrophils specializing in phagocytosis and oxidative killing and *cpa5* ^+^ granulocytes involved in immunomodulation and pathogen recognition.

Granulocytes were present in limited numbers in the gills of healthy fish (neutrophils: 2.31%; *cpa5* ^+^ granulocytes: 0.86%) (**Figure 7B**). After *C. irritans* challenge, neutrophil counts increased to 5.07% (a 2.2-fold increase), while *cpa5* ^+^ granulocyte counts remained stable (**Figure 7B**), suggesting the pathogen-specific mobilization of phagocytic defenses. This stability suggests that *cpa5* ^+^ granulocytes may act as tissue-resident sentinels or immunomodulators, rather than undergoing rapid recruitment like neutrophils, potentially providing sustained regulatory signals or acting as a reservoir for later effector responses. Infection-responsive transcriptomic shifts revealed metabolic activation in both lineages, with upregulated oxidative phosphorylation (ko00190) The pathway Ribosome, which extends beyond the scope presented in **Figure 7C** (top 10), is not displayed in **Figure 7C**, despite the presence of significant enrichment. Therefore, we have removed this pathway, and Proteasome (ko03050) pathways (**Figures 7C, D**). The infection induced upregulation of chemoattractant (*cklf*) and respiratory burst effectors (*rac2* and *mpx*), while suppressing inflammatory mediators (*il8*, *tlr5*, and *clec4el*) (**Figure 7E**). This dual response may indicate feedback regulation to mitigate tissue damage during late infection phases.

### 3.8 The responses of macrophages, dendritic cells and B cells in gills to *C. irritans* infection

**Figure 8:**
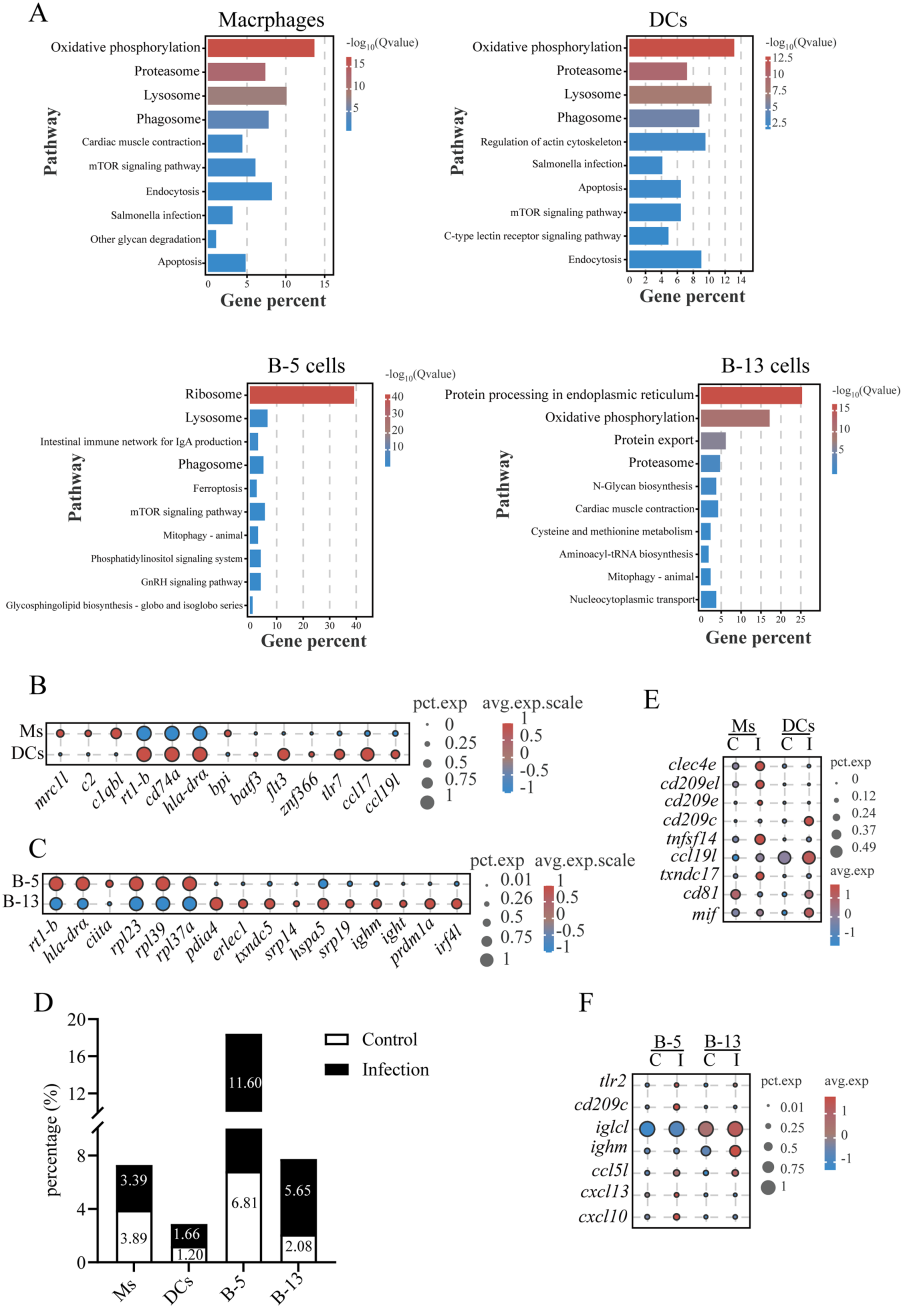
Characterization of macrophages, dendritic cells and B cells in gills and their responses to *C*. *irritans* infection. **(A)** KEGG pathway enrichment in macrophages, DCs, B-5 cells and B-13 cells. **(B)** Bubble plot comparing differentially expressed genes (DEGs) between macrophages (Ms) and DCs, highlighting lineage-specific functional difference. **(C)** Dot plot of differentially expressed genes between B-5 cells and B-13 cells. **(D)** Macrophages, DCs, B-5 cells and B-13 cells proportions in control vs. infected groups. **(E)** Immune-related gene expression changes in macrophages (Ms) and DCs post-infection. **(F)** Immune-related gene expression changes in B-5 cells and B-13 cells post-infection.

KEGG pathway analysis revealed an upregulation of genes related to lysosomal and phagosomal pathways in macrophages, DCs, and B-5 cells (Cluster 5) (**Figure 8A**). Key genes within these pathways included cathepsins (*ctsb*, *ctsk*, and *ctsz*), lysosomal membrane proteins (*limp2*, *npc2*, and *laptm4a*), glycosidases (*hexb*), vacuolar ATPase subunits (*atp6s1*, *atp6l*, and *atp6n*), NADPH oxidase components (*ncf1*, *nox2*, and *cyba*), and vesicular trafficking regulators (*rab5c*, *rab7*, and *rab11b*) (**Supplementary Table S14**). These data suggest that B-5 cells, similar to macrophages and DCs, have phagocytic capacity. Macrophages expressed lineage-specific receptors (e.g., *marco*, *mrc1*, and *csf1rb*), complement system components (e.g., *c1qbl*, *c2*, and *cfb*), antigen presentation molecules (e.g., *hla-drα*, *rt1-b*, and *cd74*), and antimicrobial protein gene (*bpi*), indicating their roles in complement activation, antigen processing/presentation, and pathogen clearance in the gills (**Figures 8B**, **1B**). DCs were characterized by transcription factors essential for their development (*flt3*, *batf3*, and *znf366*) (37), pattern recognition receptor (*tlr7*- a viral ssRNA sensor) (38), chemokines (*ccl17* and *ccl19l*), and antigen presentation machinery (e.g., *hla-drα*, *rt1-b*, and *cd74*) (**Figure 8B**). B-5 cells exhibited antigen presentation activity (e.g., *hla-drα*, *rt1-b*, and *ciita*) and ribosome biogenesis signatures (e.g., *rpl23*, *rpl39*, and *rpl37a*) (**Figure 8C**), similar to B1-like cells in zebrafish (39). In contrast, B-13 cells showed features of plasma cells, with an enrichment in ER stress pathway activation (e.g., *pdia4*, *erlec1*, and *txndc5*), and upregulation of protein export machinery (e.g., *srp14*, *hspa5*, and *srp19*), immunoglobulin synthesis (*ighm* and *ight*), and differentiation-driving transcription factors (*irf4l* and *prdm1a*) (40) (**Figure 8C**).

After *C. irritans* infection, the abundance of DCs, B-5, and B-13 cells increased from 1.20% to 1.66%, 6.81% to 11.60%, and 2.08% to 5.65%, respectively, while macrophages decreased (from 3.89% to 3.39%) (**Figure 8D**). Results found upregulation of pathogen-recognition receptors (*clec4e*, *cd209c*, *cd209e*, and *tlr2*) in macrophages, DCs, or B-5 cells in infected fish compared to controls (**Figures 8E, F**). Macrophages in the gills of infected fish exhibited increased expression of *tnfsf14* (a cytokine that promotes T cell proliferation (41), 8.64-fold), *ccl19l* (a chemokine critical for naïve T cell recruitment (42), 3.37-fold), and *txndc17* (a redox homeostasis regulator (43), 4.22-fold), compared to the controls. DCs showed elevated expression of *tnfsf14* (15.06-fold), *cd81* (MHC-II compartment regulator, 4.58-fold) and *mif* (a macrophage migration inhibitor, 3.7-fold) in infected fish compared to controls. Both B-5 and B-13 cells exhibited enhanced antibody production (*iglcl* and *ighm*) and upregulated expression of the T cell chemoattractant (*ccl5l*) in the infected fish (**Figure 8F**). Notably, B-5 cells specifically upregulated a lymphoid-organizing chemokine responsible for B cell follicle formation (44) (*cxcl13*) and a Th1-associated chemokine mediating T/NK cell recruitment (*cxcl10*) in the infected fish (**Figure 8F**). These results suggest coordinated roles for B cell subsets in adaptive immune regulation and lymphoid tissue remodeling in the gills of *L. crocea* during *C. irritans* infection.

## 4 Discussion

As a multifunctional organ at the host-environment interface, the gills of teleosts balance physiological homeostasis with pathogen defense through specialized leukocyte networks. However, their composition, heterogeneity, and regulatory roles in immune homeostasis remain largely unknown. T cells are crucial mediators of adaptive immunity. Our scRNA-seq analysis reveals that T cells dominate (>70%) the immune cell landscape of *L. crocea* gills, consistent with reports in *Scophthalmus maximus* and *Oreochromis niloticus* (10, 11). Similarly, in zebrafish (*Danio rerio*), recent immunohistochemical studies demonstrated an abundance of T cells in gill mucosa-associated lymphoid tissues (45, 46). These results indicate the important role of T cells in teleost gill immunity. Our results further identified seven T cell subpopulations in the gills, including DNTs, CD4^+^ naïve T cells, CD4^+^ Foxp3^+^ Tregs, CD4^+^ Th17 cells, CD4^+^ Th1 cells, CD8^+^ T cells, and γδ T cells, similar to the case of *S. maximus* gills (11). Critically, all *L. crocea* T cell subsets co-expressed TCR signaling components (*zap70*, *lck*, and *cd3ζ*), confirming the evolutionary conservation of antigen-driven activation mechanisms. These findings demonstrate that teleost gills contain multiple functionally specialized T cell lineages, which may play important roles in gill immunity.

The differentiation of naïve CD4^+^ T cells into specialized effector subsets represents a cornerstone of adaptive immunity, with lineage commitment dictated by pathogen class (47–50). Our study revealed that *C. irritans* infection elicits minimal Th1/Th17 subset modulation in *L. crocea* gills, a phenomenon potentially attributable to a shorter duration of infection or parasite-specific immunomodulation. Nevertheless, localized T cell proliferation acceleration aligns with prior reports of *C. irritans* infection causing clonal expansion of T cells in teleost gills (23). Notably, *C. irritans* infection triggered Treg differentiation and activation, characterized by the upregulation of *foxp3* and *tgfb1*. This is consistent with mammalian helminth infection models, in which parasites directly or indirectly drive Treg differentiation through TGFβ signaling (51). Treg expansion likely represents a host defense mechanism to limit infection-induced immunopathology, such as tissue damage. However, it may also represent a strategy exploited by *C. irritans* to dampen effective anti-parasitic immunity and facilitate persistence. Excessive Treg activity can impair parasite clearance, as demonstrated in mouse models where Treg depletion enhanced pathogen elimination (52, 53). These findings suggest that *C. irritans* may exploit Treg expansion as an immune evasion strategy. Manipulating Treg activity, such as via targeted inhibition of TGFβ signaling during early infection, represents a potential therapeutic strategy for enhancing parasite clearance in aquaculture settings.

In contrast to mammalian CD8^+^ CTLs, which predominantly mediate antigen-dependent elimination of virus-infected or malignant cells through MHC-I-restricted mechanisms, teleost CD8^+^ T cells exhibit innate-like cytotoxicity against a variety of pathogens, including extracellular parasites. For example, *Carassius auratus* CD8^+^ T cells directly lyse *Ichthyophthirius multifiliis* depending on serine proteases and perforin (54). The cytolytic machinery may involve perforin-mediated pore formation, facilitating granzymes (serine proteases) delivery to disrupt target cell membrane integrity. Similarly, the antimicrobial peptide Nk-lysin (NKL, encoded by *nkl*) in teleosts demonstrates anti-parasitic activity (55). In *L. crocea*, transcriptional upregulation of *nkl*, *prf1*, and *gzmb* in CD8^+^ CTLs during *C. irritans* infection suggests conserved cytotoxic pathways against ectoparasites. This functional difference highlights the evolutionary ancient role of cytotoxic lymphocytes in direct anti-parasitic defense, with innate-like mechanisms operating alongside MHC-restricted immunity, emphasizing the importance of such responses on the mucosal surfaces of teleosts.

Despite sharing regulatory functions with Tregs, DNTs also secrete cytokines (IL-4, IL-17, IFN-γ, and TNF-α) to exert T helper (Th)-like activities, exhibiting protective roles across infection models in mammals (27). For instance, DNTs constitute a major responding T cell subset in murine lungs during *Francisella tularensis* infection, conferring protection *via* IL-17A and IFN-γ production (56). In teleosts, DNTs in *S. maximus* showed upregulated expression of cytotoxicity-related genes (*faslg*, *nkl3*, *gzma*, *gzmb*, and *prf1*) and the immunoregulatory gene *il10* during *Edwardsiella piscicida* infection, suggesting dual roles in cytolysis and immune modulation (11). Our findings revealed that *Lc*DNTs displayed Th17-like functional program (*rorc* and *il17a/f1*) and upregulated immunomodulatory factors (*ccl5l* and *tnf*) following *C. irritans* challenge. These results indicate that DNTs in teleosts, like mammals, contribute to the inflammatory response while retaining regulatory potential, underscoring the evolutionary conservation of the multifunctional role of DNTs in mucosal immunity.

γδ T cells represent an evolutionarily primitive T cell subset characterized by distinct TCRs and roles in innate and adaptive immunity, mainly categorizing into two functionally distinct T cell subsets (IFN-γ-producing γδ T1 cells and IL-17-secreting γδ T17 cells) (57). Additionally, Type 2-like γδ T effector cells have been identified in humans, although they do not consistently express signature cytokines such as IL-4 (58). In addition, a specific subset of V_γ_1^+^V_δ_6^+^ T cells in mice can produce large amounts of IL-4 and participate in helping B cells produce IgE (59). In teleosts, γδ T cells with functional similarities to mammalian γδ T cells have been found. For example, γδ T cells in *D. rerio* exhibit potent phagocytic capabilities and serve as antigen-presenting cells, initiating CD4^+^ T cell activation and promoting IgM/IgZ production (60). However, Th-like γδ T cells (specialized type 2 effectors) have not yet been identified in teleosts. In this study, γδ T cells in *L. crocea* displayed Th2-like effector properties, as marked by *il4/13a/b* expression. This reports the first characterization of specialized type 2 effector γδ T cells in teleosts, revealing a previously unrecognized heterogenous class within this lineage and a potential important role in regulating humoral immunity (e.g., Ig production) against parasitic infections.

ILC2s serve as tissue-resident sentinels in mucosal tissues, modulating type 2 immunity through GATA3-dependent cytokine production (e.g., IL-4/IL-13) to combat extracellular parasites (31, 36, 61). The identification of *Lc*ILC2s expressing *gata3*, *il4/13a/b*, and *st2* (IL-33R) provided the first evidence of a conserved type 2 immune axis in the gills of teleost fish. The increased number of *Lc*ILC2s (8.7-fold) and cytokine induction in infected fish, compared with controls, are similar to the mammalian ILC2s response to helminths (62), suggesting its important role in anti-parasitic defenses and highlighting ILC2s as potential targets for mucosal vaccines against ectoparasites like *C.irritans*. In mammals, epithelial alarmins (IL-25/IL-33) activate ILC2s *via* receptors (IL-25R/IL-33R), driving IL-4/IL-13-dependent macrophage polarization, Th2 differentiation, and epithelial remodeling to expel parasites (50, 63–65). Intriguingly, the absence of canonical ILC2-activating receptors (*il7r/il25r*) and the unique expression of *il21rl* suggest a potential teleost-specific regulatory difference. Therefore, we propose that IL-21, a pleiotropic type I cytokine that modulates multiple immune cell types (T/B cells, macrophages, and DCs) (66), may serve as a potential teleost-specific ILC2 regulator and an immunostimulant against parasitic infection, although this hypothesis requires further confirmation. Notably, compared to conventional CD4^+^ T cells, the expression levels of *gata3*, *il4/13a*, and *il4/13b* are higher in *Lc*ILC2s, emphasizing the crucial role of ILC2s in the type 2 immune response of fish gills, especially in anti-parasitic defenses. This functional conservation of type 2 effector mechanisms, coupled with different receptor utilization, highlights both shared ancestry and lineage-specific adaptations in vertebrate ILC2 biology. These findings establish teleost ILC2-like cells as ancient mediators of mucosal antiparasitic immunity.

The phagocytic capacity of B-5 cells, marked by enrichment in phagosomal and lysosomal pathways, is consistent with emerging evidence that teleost B cells contribute to pathogen clearance *via* phagocytosis (67, 68). The higher abundance of both B-5 and plasma cell-like B-13 cells, combined with enhanced expression of *ighm* in infected gills, directly correlates with findings showing increased levels of parasite-specific IgM locally in the gills of *L. crocea* after *C. irritans* infection (23). This confirms the functional contribution of these gill-resident B cell subsets to local mucosal antibody responses. Recent advances underscore the significance of TLRs (e.g., TLR2) as pivotal pathogen sensors in aquatic species (69). Supporting this, our data show upregulation of *tlr2* in infected B-5 cells, indicating its potential involvement in anti-parasitic responses. In mice, CXCL13 is a crucial regulator of B-cell migration and lymphoid tissue architecture, with CXCL13^−/−^ mice exhibiting aberrant follicular organization (44). By analogy, the upregulation of *cxcl13* in B-5 cells may promote the formation of gill-associated lymphoid tissues (e.g., ILT and ALT) in the gills of *L. crocea*. In *Paralichthys olivaceus*, CXCL10 exhibits dual functions as both an immunoregulator and a bactericidal agent (70). Thus, the induction of *cxcl10* in B-5 cells may represent an additional mechanism to combat parasites, which needs confirmation in further studies. DCs emerged as important coordinators of adaptive immunity, enhancing *tnfsf14* expression to promote T cell proliferation and *ccl19l* expression to facilitate naïve T cell recruitment. This functional regulation of DCs on T cells underscores their conserved role in bridging innate and adaptive defenses across vertebrates.

**Figure 9:**
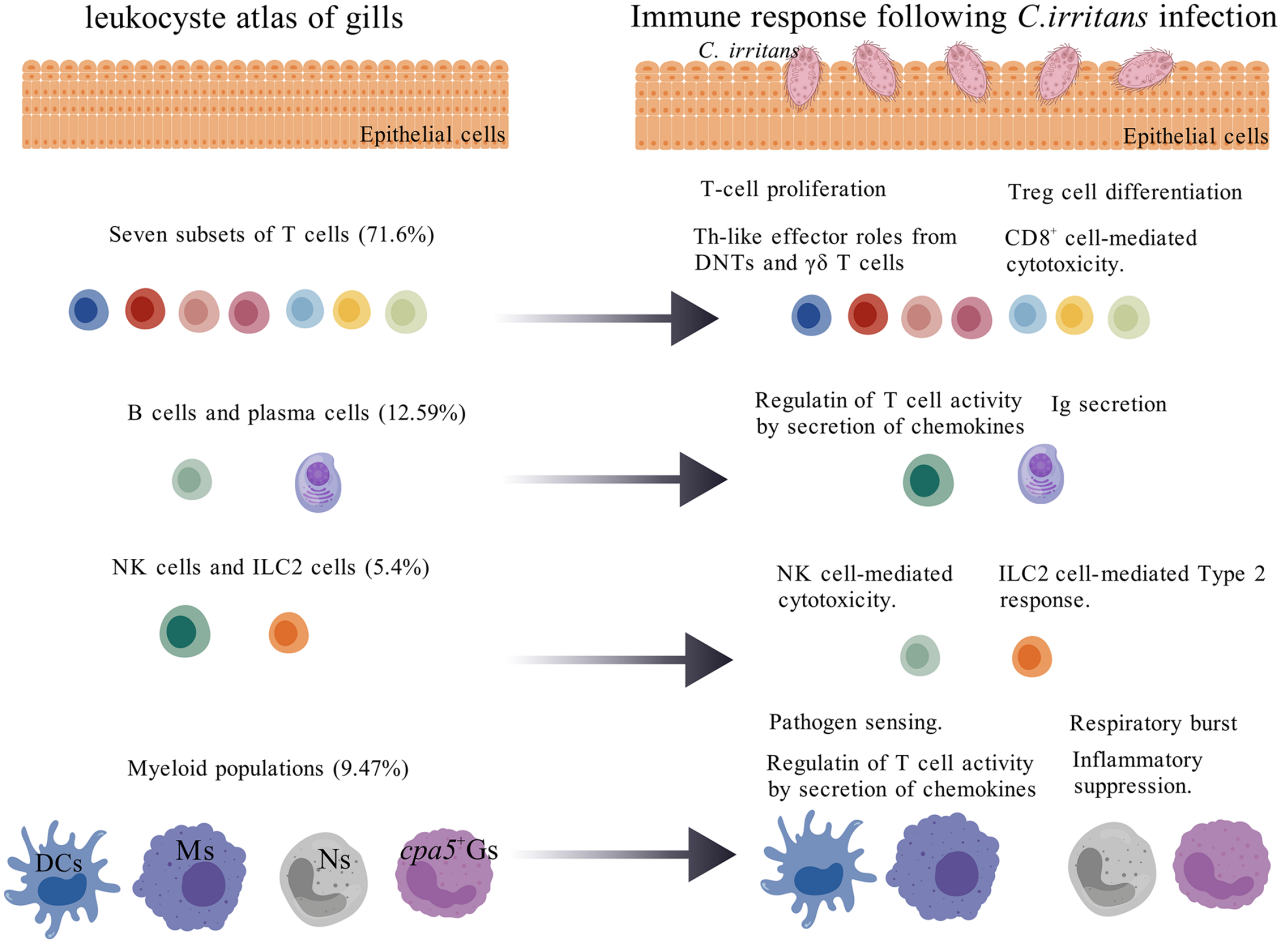
Leukocyte heterogeneity in the gills of *L. crocea* in response to *C. irritans* infection. Gill leukocytes are predominantly composed of T cells (>70% of total immune cells), followed by B cells, myeloid cells, and innate granulocytes (NK-like cells and ILC2-like cells). *C. irritans* infection triggered T cell proliferation and functional diversification, including: regulatory T cell (Treg) differentiation (Foxp3^+^), cytotoxic CD8^+^ T cell activation, Th17-like activity in DNTs, and Th2-like responses in γδ T cells. B cells exhibited IgM secretion and chemokine production. ILC2-like cells expanded significantly (8.7-fold increase) with upregulated type 2 cytokine expression, while cytotoxic NK subsets enhanced effector functions. Neutrophil (Ns) abundance and oxidative burst activity increased, whereas *cpa5* ^+^ granulocytes (*cpa5* ^+^Gs) maintained stable abundance but promoted immunomodulation. Macrophages (Ms) and dendritic cells (DCs) adopted compartment-specific activation states, upregulating gene modules linked to pathogen sensing, antigen processing/presentation, and chemotaxis.

In conclusion, this study systematically deciphered the cellular atlas of mucosal immunity in the gills of teleost fish (**Figure 9**), unveiling a multi-layered defense mechanism mediated by various immune cells and providing an important model for comparative immunology. Future research may incorporate additional methodologies, such as cell sorting, CRISPR screening, or cell depletion, to further validate the role of the IL21RL signaling pathway in ILC2s and the mechanisms of Treg-mediated immune tolerance, providing new targets for anti-parasitic strategies in aquaculture.

## Data Availability

The datasets presented in this study can be found in online repositories. The names of the repository/repositories and accession number(s) can be found below: https://ngdc.cncb.ac.cn/gsa/, CRA025829.
