# Supplementary material for: Single-cell transcriptomics unveils leukocyte heterogeneity in the gills of Larimichthys crocea in response to parasitic infection
Source: Front Immunol. 2025 Aug 1;16:1633701. doi: 10.3389/fimmu.2025.1633701 (PMC12353724; doi:10.3389/fimmu.2025.1633701)
Supplement: Supplementary file 1 [file DataSheet1.pdf]

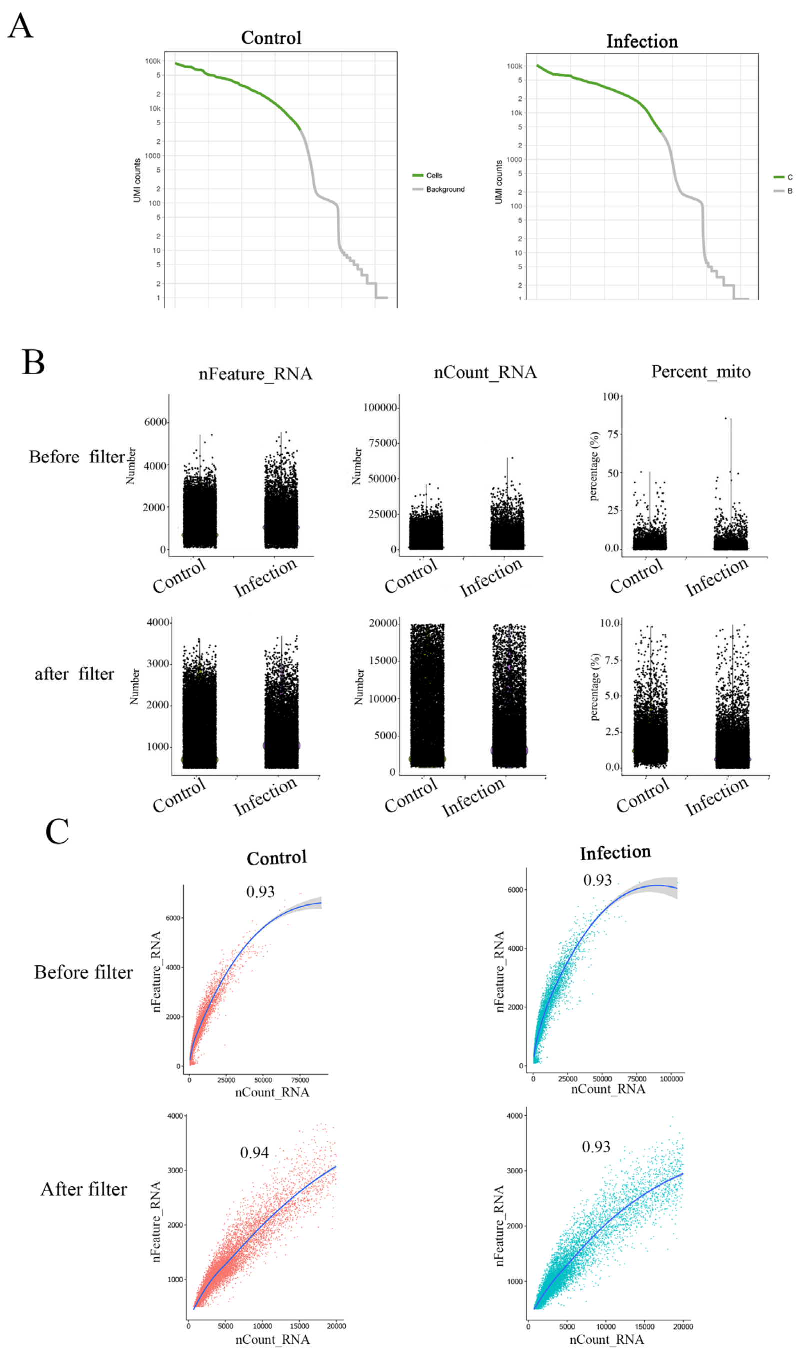

Supplementary Figure 1. Single cell sequencing quality control. (A) Identification of the effective cell number of the sample. The X_axis is the number of barcodes, and the Y_axis is the UMI counts. Green line is the effective cells corresponding to barcodes, and the gray line is the background noise. (B) The basic information of sample cell before and after filtering, including the total number of genes (nFeature_RNA), the total number of UMIs (nCount_RNA) and the percentage of reads mapping mitochondrial genes (Percent.mito). (C) The scatter plot of sample cell basic information before and after filtering, including the relationship between nCount_RNA and nFeature_RNA, and between nCount_RNA and percent_mito. The numbers above the graph are Pearson correlation coefficients.

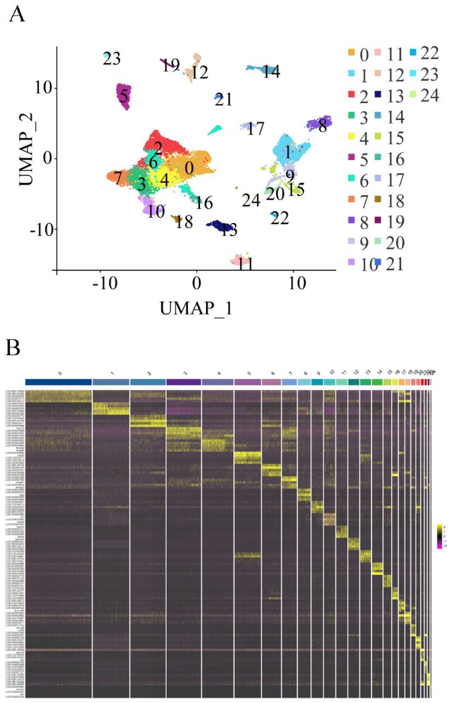

Supplementary Figure 2. (A) A total of 25 cell clusters were identified and shown with a uniform manifold approximation and projection (UMAP) space. (B) Heatmap of marker genes from each cluster. Each column in the figure represents a cell subtype, and each row represents a gene. The expression levels of genes in different cells are indicated by different colors. An intensely yellow color indicates high expression, and an intensely purple indicates low expression.

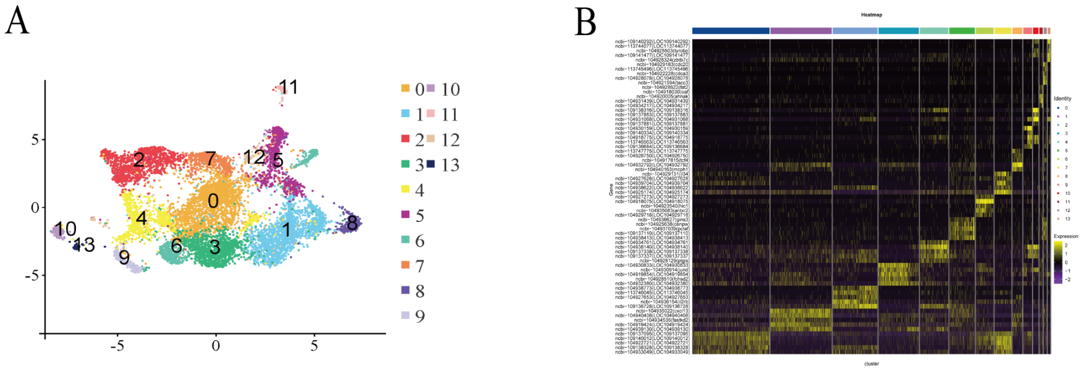

Supplementary Figure 3. (A) A total of 14 cell clusters for T cells were identified and shown with a uniform manifold approximation and projection (UMAP) space. (B) Heatmap of marker genes from each cluster. Each column in the figure represents a cell subtype, and each row represents a gene. The expression levels of genes in different cells are indicated by different colors. An intensely yellow color indicates high expression, and an intensely purple indicates low expression.

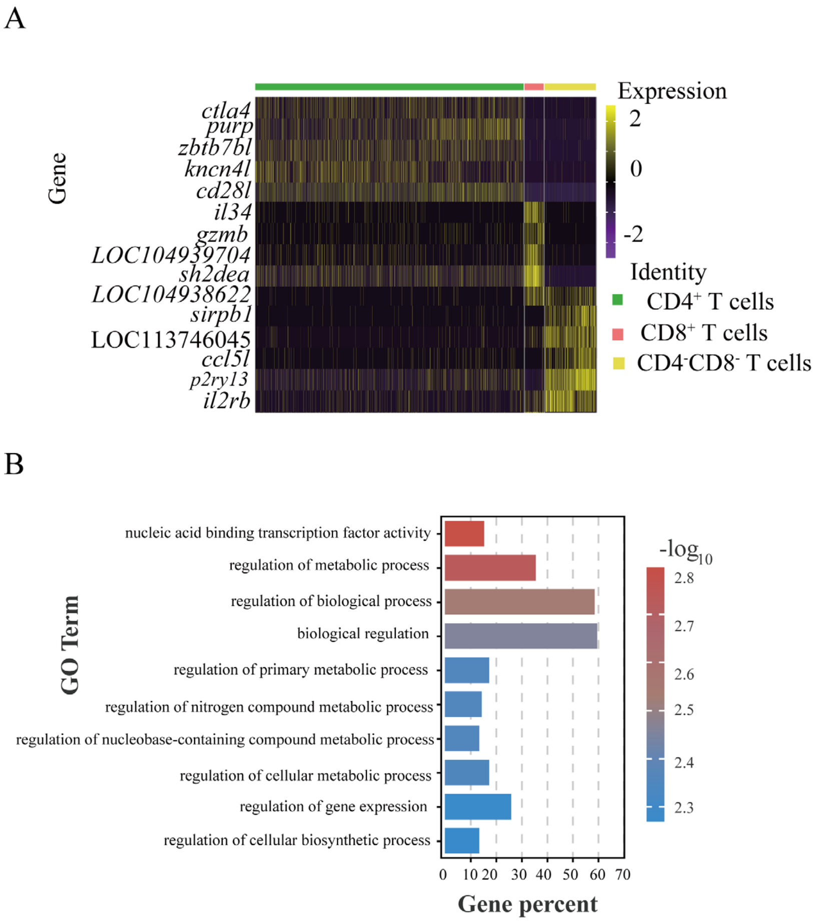

Supplementary Figure 4. (A) Heatmap of the top 5 most differently expressed genes for each T cell subset. (B) GO enrichment analysis of genes in CD4$^-$CD8$^-$ T cells.

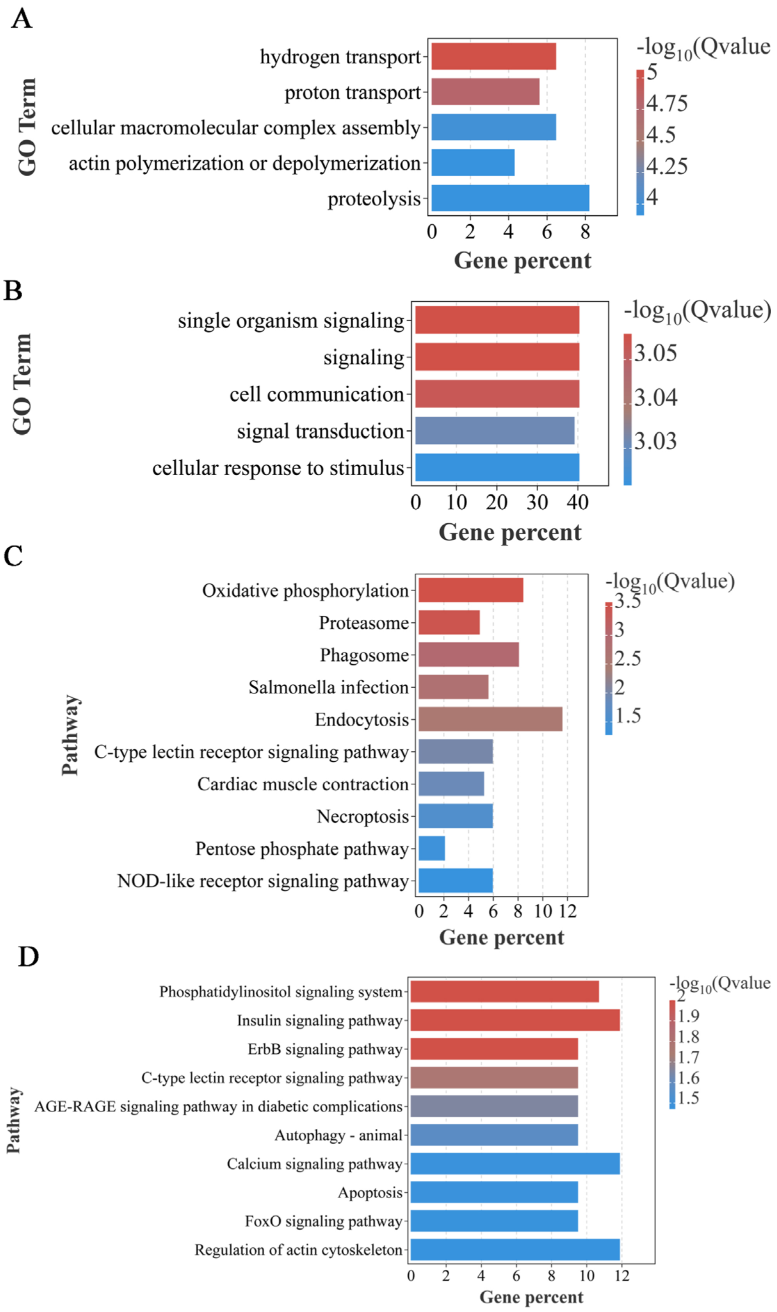

Supplementary Figure 5. GO enrichment analysis of genes in neutrophils (A) and *cpa5*+ granulocytes (B). KEGG enrichment analysis of neutrophils (C) and *cpa5*+ granulocytes (D).
